# Molecular Identification and Genotyping of *Cryptosporidium* spp. and *Blastocystis* sp. in Cattle in Representative Areas of Shanxi Province, North China

**DOI:** 10.3390/ani13182929

**Published:** 2023-09-15

**Authors:** Yao Liang, Ya-Ya Liu, Jin-Jin Mei, Wen-Bin Zheng, Qing Liu, Wen-Wei Gao, Xing-Quan Zhu, Shi-Chen Xie

**Affiliations:** 1 Laboratory of Parasitic Diseases, College of Veterinary Medicine, Shanxi Agricultural University, Taigu, Jinzhong 030801, China; liangyao2219@163.com (Y.L.); ly1397772866@163.com (Y.-Y.L.); mjj3051@163.com (J.-J.M.); wenbinzheng1@126.com (W.-B.Z.); lqsxau@163.com (Q.L.); sxndgaowenwei@163.com (W.-W.G.); 2 Research Center for Parasites & Vectors, College of Veterinary Medicine, Hunan Agricultural University, Changsha 410128, China; 3 Key Laboratory of Veterinary Public Health of Higher Education of Yunnan Province, College of Veterinary Medicine, Yunnan Agricultural University, Kunming 650201, China

**Keywords:** *Cryptosporidium* spp., *Blastocystis* sp., genotypes, subtypes, prevalence, cattle, Shanxi Province

## Abstract

**Simple Summary:**

*Cryptosporidium* spp. and *Blastocystis* sp. are zoonotic intestinal protozoa, which cause intestinal diseases in humans and animals. So far, the prevalence of these two parasites in cattle in Shanxi Province remains unknown. Thus, the present study collected 795 fecal samples from cattle in Shanxi Province and these fecal samples were examined for presence of *Cryptosporidium* spp. and *Blastocystis* sp. using molecular methods. The prevalence of *Cryptosporidium* spp. in cattle in Shanxi Province was 2.9% (23/795), and all of the positive samples were identified as *C. andersoni*. The prevalence of *Blastocystis* sp. in cattle in Shanxi Province was 13.0% (103/795), including five known subtypes (ST1, ST10, ST14, ST21 and ST26) and three unknown subtypes. Furthermore, the genetic diversity and distribution of *Cryptosporidium* spp. and *Blastocystis* sp. were further explored through the analysis of DNA sequences and phylogenetic trees. This study reveals the presence and prevalence of *Cryptosporidium* spp. and *Blastocystis* sp. in cattle in Shanxi Province for the first time, and provides baseline data for the control and prevention of both parasites in cattle in Shanxi Province.

**Abstract:**

Both *Cryptosporidium* spp. and *Blastocystis* sp. are common intestinal protozoa, which can cause zoonotic diseases and economic losses to livestock industry. To evaluate the prevalence and genetic population structure of *Cryptosporidium* spp. and *Blastocystis* sp. in beef and dairy cattle in Shanxi Province, north China, a total of 795 fecal samples were collected from beef and dairy cattle in three representative counties in Shanxi Province, and these fecal samples were examined using molecular approaches based on 18S small-subunit ribosomal RNA (SSU rRNA) of *Cryptosporidium* spp. and *Blastocystis* sp., respectively. Among 795 cattle fecal samples, 23 were detected as *Cryptosporidium*-positive and 103 were detected as *Blastocystis*-positive, and the overall prevalence of *Cryptosporidium* spp. and *Blastocystis* sp. in cattle in Shanxi Province was 2.9% and 13.0%, respectively. For *Cryptosporidium* spp., DNA sequence analysis indicated that all 23 positive samples were identified as *C. andersoni*. Furthermore, five known subtypes (ST1, ST10, ST14, ST21 and ST26) and three unknown subtypes of *Blastocystis* sp. were detected among 103 positive samples using DNA sequence analysis. This study reported the occurrence and prevalence of *Cryptosporidium* spp. and *Blastocystis* sp. in cattle in Shanxi Province for the first time, which extends the geographical distribution of these two zoonotic parasites and provides baseline data for the prevention and control of these two important zoonotic parasites in cattle in Shanxi Province.

## 1. Introduction

*Cryptosporidium* spp. is one of the six major pathogens causing diarrheal diseases in ruminants, and can also cause gastrointestinal diseases in a variety of vertebrate hosts including domestic animals, wild animals and humans [1,2,3]. The life cycle of *Cryptosporidium* spp. consists of five developmental stages: trophozoites, schizophytes, gametophytes, zygotes and oocysts, of which the oocysts excreted with the host’s feces are infectious [4]. Up to now, 44 species of *Cryptosporidium* spp. and more than 120 genotypes have been reported worldwide [5]. A large amount of research has indicated that *C. andersoni*, *C. parvum*, *C. bovis* and *C. ryanae* are the main causative agents of cryptosporidiosis in cattle [5,6]. In addition, *C. andersoni*, *C. parvum* and *C. bovis* can infect not only cattle but also humans [5]. Cryptosporidiosis is highly correlated with the immune status of the host [7]. Normally, the typical symptoms, such as dehydration and diarrhea, occur in cattle following infection with *Cryptosporidium* spp., and abdominal pain or vomiting have been occasionally reported in very few cattle.

*Blastocystis* sp. is an anaerobic zoonotic parasite found in most mammals and birds in many countries [8,9]. The most typical morphotype of *Blastocystis* sp. is the vacuolar form, which is also the most common form in cattle [10]. At present, increasing studies indicate that *Blastocystis* sp. isolates detected in different hosts around the world can be divided into 32 subtypes (STs) according to the SSU rRNA sequences of *Blastocystis* sp. [11]. Among these subtypes, ST1-9, ST12 and ST24 were mostly presented in humans, of which ST1-4 and ST3 were the mostly prevalent subtypes in humans and children with diarrhea, respectively [12]. Subtypes ST10, ST11 and ST13-17 were mainly detected in animals, ST1-7, ST10 and ST12-15 were generally identified in ungulates, and ST1, ST2, ST4 and ST6-8 were reported in birds [13]. The latest epidemiological surveys indicated that *Blastocystis* sp. has infected one to two billion people around the world [14,15]. Cattle, one of the most common hosts of *Blastocystis* sp., were usually asymptomatic after infection with *Blastocystis* sp., whereas diarrhea, abdominal pain and other gastrointestinal symptoms might occur in immunocompromised individuals [16].

Shanxi Province is an agricultural province combining mountains and rivers in north China. Its rich natural grassland and crop straw resources have created superior conditions for the development of high-quality beef cattle and dairy cows. Infection with *Cryptosporidium* spp. and *Blastocystis* sp. may not only affect the health of cattle in Shanxi Province, but also the health of humans and other animals through transmission via the fecal–oral route, resulting potential public health problems. Cattle are considered important hosts for both *Cryptosporidium* spp. and *Blastocystis* sp., and there have been no relevant reports about the infection of both parasites in cattle in Shanxi Province until now. Thus, the objectives of the present study were to investigate the prevalence and genetic structure of *Cryptosporidium* spp. and *Blastocystis* sp. in cattle in Shanxi Province, north China.

## 2. Materials and Methods

### 2.1. Study Design and Sample Size

This study used a cross-sectional study design with a simple random sampling procedure to assess the prevalence of *Cryptosporidium* spp. and *Blastocystis* sp. in representative areas of Shanxi Province. The sample size was determined using the formula given by Thrusfield [17], with the 95% confidence intervals and 5% precision value.

$$n=\frac{1.96^{2}\times P_{exp}\left(1-P_{exp}\right)}{d^{2}}$$

*n* = the required sample size;
*P_exp_* = expected prevalence;
*d* = desired absolute precision.

Based on the reported prevalence of *Cryptosporidium* spp. in cattle in China, the expected prevalence was taken as 11.9% [18]. The sample size was calculated as 161 of each study area using the above formula.

### 2.2. Fecal Sample Collection and Preparation

**Figure 1:**
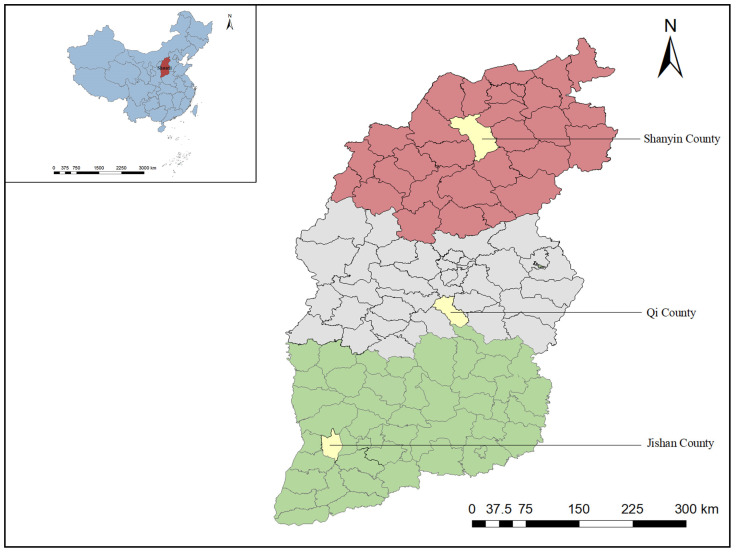
A map showing the sampling sites in Shanxi Province in this study. The map was made by ArcGIS10.8 and based on data from the Resource and Environmental Science and Data Center of the Chinese Academy of Sciences. The color of red, gray and green in the map of Shanxi Province represent the northern, central and southern regions of Shanxi Province, respectively.

In November 2020, 795 fecal samples of beef cattle (401) and dairy cattle (394) were collected from three representative counties located in northern, central and southern Shanxi Province (34°34′–40°44′ N, 110°14′–114°33′ E). As shown in Figure 1, a total of 209, 313 and 273 cattle fecal samples were collected from Shanyin County, Qi County and Jishan County, respectively. All fecal samples were obtained from captive cattle, with 525 female cattle and 270 male cattle. Sampled cattle feces were collected from three age groups, including 286 cattle younger than 12 months, 195 cattle aged 12–18 months, and 314 cattle older than 18 months. With the consent of the owner, all fecal samples were collected from the top center of fresh feces freely excreted by the cattle to ensure no contamination. Each sample was individually packaged to prevent cross-contamination between samples. The packaged samples were briefly stored in a low-temperature environment, and transported to the laboratory and stored at −20 °C.

### 2.3. DNA Extraction and PCR Amplification

For each fecal sample, approximately 200 mg mixed feces was used to extract the genomic DNA by using the Omega E.Z.N.A.^®^ Stool DNA kit (Omega Bio-tek, Norcross, GA, USA) following the manufacturer’s instruction, and the extracted genomic DNA was stored in a freezer at −20 °C until PCR amplification. Subsequently, the prevalence and genotypes of *Cryptosporidium* spp. and *Blastocystis* sp. in cattle were examined via PCR targeting the 830 bp region of the SSU rRNA gene and the 613 bp region of the SSU rRNA gene, respectively [19,20]. The primers used in this study are summarized in Table 1. The 25 μL PCR mixture contained 2.5 μL of 10×PCR Buffer (Mg^2+^ free), 2 μL of dNTPs, 25 mM of MgCl_2_, 1.25 U of *Ex*-Taq, 1 μM of each primer, 2 μL of DNA samples and 14.75 μL of ddH_2_O. The reagents used in PCR amplification were purchased from TaKaRa Biotech Ltd. (Dalian, China). The reaction conditions and parameters for the amplification of the SSU rRNA gene from *Cryptosporidium* spp. were as follows: initial denaturation (94 °C for 5 min); denaturation (94 °C for 30 s), annealing (56 °C for 30 s for the primary PCR and 58 °C for 30 s for the secondary PCR) and extension (72 °C for 30 s) for 35 cycles; and final extension (72 °C for 10 min). For amplification of the SSU rRNA gene of *Blastocystis* sp., the PCR conditions were as follows: initial denaturation (95 °C for 3 min); denaturation (94 °C for 1 min), annealing (65 °C for 1 min) and extension (72 °C for 1 min) for 35 cycles; and final extension (72 °C for 2 min). The positive control (DNA of each parasite previously verified by sequencing) and negative control (reagent-grade water) were added to each PCR round to ensure the reliability of the results. The PCR products were examined using gel electrophoresis in 2% agarose with ethidium bromide, and the positive products were sent to Sangon Biotech Ltd. (Shanghai, China) for two-directional sequencing.

### 2.4. Sequencing and Phylogenetic Analysis

In this study, the obtained DNA sequences of *Cryptosporidium* spp. and *Blastocystis* sp. were proofread and edited with Chromas Pro v2.1.3 software. On the National Center for Biotechnology Information website (https://www.ncbi.nlm.nih.gov/, accessed on 6 August 2020), the Basic Local Alignment Search Tool (BLAST) was applied to align the DNA sequences with the reported DNA sequences of *Cryptosporidium* spp. and *Blastocystis* sp. to determine the genotypes. The phylogenetic trees were constructed based on the Neighbor-Joining (NJ) method and the Kimura 2-parameter model using Mega 11.0, and 1000 replicates (bootstrap value) were selected to assess the robustness of the findings [14,21,22].

### 2.5. Statistical Analysis

In this study, the correlation between the prevalence and risk factors (regions, ages and genders) of *Cryptosporidium* spp. and *Blastocystis* sp. was statistically analyzed using Chi-square (χ^2^) test in software SPSS 26.0 (IBM, Chicago, IL, USA), respectively. In addition, the strength of the association between prevalence and test conditions was assessed by calculating the odds ratio (ORs) and 95% confidence interval (95% CI). The difference is considered significant when the *p*-value is <0.05.

## 3. Results

### 3.1. The Prevalence of Cryptosporidium spp. and Blastocystis sp. in Cattle in Shanxi Province

In this study, 23 out of 795 cattle fecal samples from three representative counties of Shanxi Province were screened as *Cryptosporidium*-positive using nested PCR amplification of the SSU rRNA gene, with an overall prevalence of 2.9% (Table 2). Notably, the highest prevalence of *Cryptosporidium* spp. was observed in Jishan County (7.0%, 19/273), followed by Qi County (1.3%, 4/313) and Shanyin County (0.0%, 0/209). There were significant differences in the prevalence of *Cryptosporidium* spp. in the three study areas (*p* < 0.001). The prevalence of *Cryptosporidium* spp. in male cattle (3.0%, 8/270) was higher than that in females (2.9%, 15/525), but the difference is not statistically significant (*p* = 0.933). Likewise, there was no significant difference (*p* = 0.852) in *Cryptosporidium* spp. prevalence among the age groups, because a similar prevalence was found in different age groups (Table 2). In this study, the prevalence of *Cryptosporidium* spp. in beef cattle and dairy cattle was 4.7% (19/401) and 1.0% (4/394), respectively, and the difference is statistically significant (*p* = 0.002).

Based on the amplification of the SSU rRNA gene of *Blastocystis* sp., a total of 103 *Blastocystis*-positive samples were detected, and the overall *Blastocystis* sp. prevalence in cattle in Shanxi Province was 13.0% (Table 3). Specifically, the highest prevalence of *Blastocystis* sp. was detected in Jishan County (32.2%, 88/273), significantly higher than that in Shanyin County (3.8%, 8/209) and Qi County (2.2%, 7/313). A significant difference in the prevalence of *Blastocystis* sp. was observed among the three study regions (*p* < 0.001). In addition, the prevalence of *Blastocystis* sp. was 8.9% (24/270) in male cattle and 15.0% (79/525) in female cattle, and the difference is statistically significant (*p* < 0.05). Among age groups, the highest prevalence of *Blastocystis* sp. was detected in cattle aged from 12 to 18 months (15.4%, 30/195), followed by cattle older than 18 months (12.4%, 39/314) and younger than 12 months (11.9%, 34/286); however, the differences were not statistically significant (*p* = 0.499). In addition, the prevalence of *Blastocystis* sp. in beef cattle (19.7%, 79/401) was significantly higher than that in dairy cattle (6.1%, 24/394) (*p* < 0.001).

Regarding *Cryptosporidium* spp., 4 and 19 positive samples were detected in dairy cattle in Qi County and beef cattle in Jishan County, respectively. In Shanyin County and Qi County, the prevalence of *Blastocystis* sp. in dairy cattle was 3.8% and 5.1%, respectively, and no positive fecal samples were detected for beef cattle. The prevalence of *Blastocystis* sp. in dairy cattle and beef cattle in Jishan County was 35.3% and 18.4%, respectively. The prevalence of *Blastocystis* sp. in female cattle (44.1%) was higher than that in males (18.8%), and there were no significant differences in age distribution. Furthermore, only 1.5% of the beef cattle (6/401) were detected as positive for co-infection of both *Cryptosporidium* spp. and *Blastocystis* sp. in Jishan County. Among them, one was male and five cattle were female, and all cattle were aged over 12 months (Table 4).

### 3.2. Distribution of Genetic Diversity and Phylogenetic Analysis of Cryptosporidium spp. and Blastocystis sp. in Cattle in Shanxi Province

**Figure 2:**
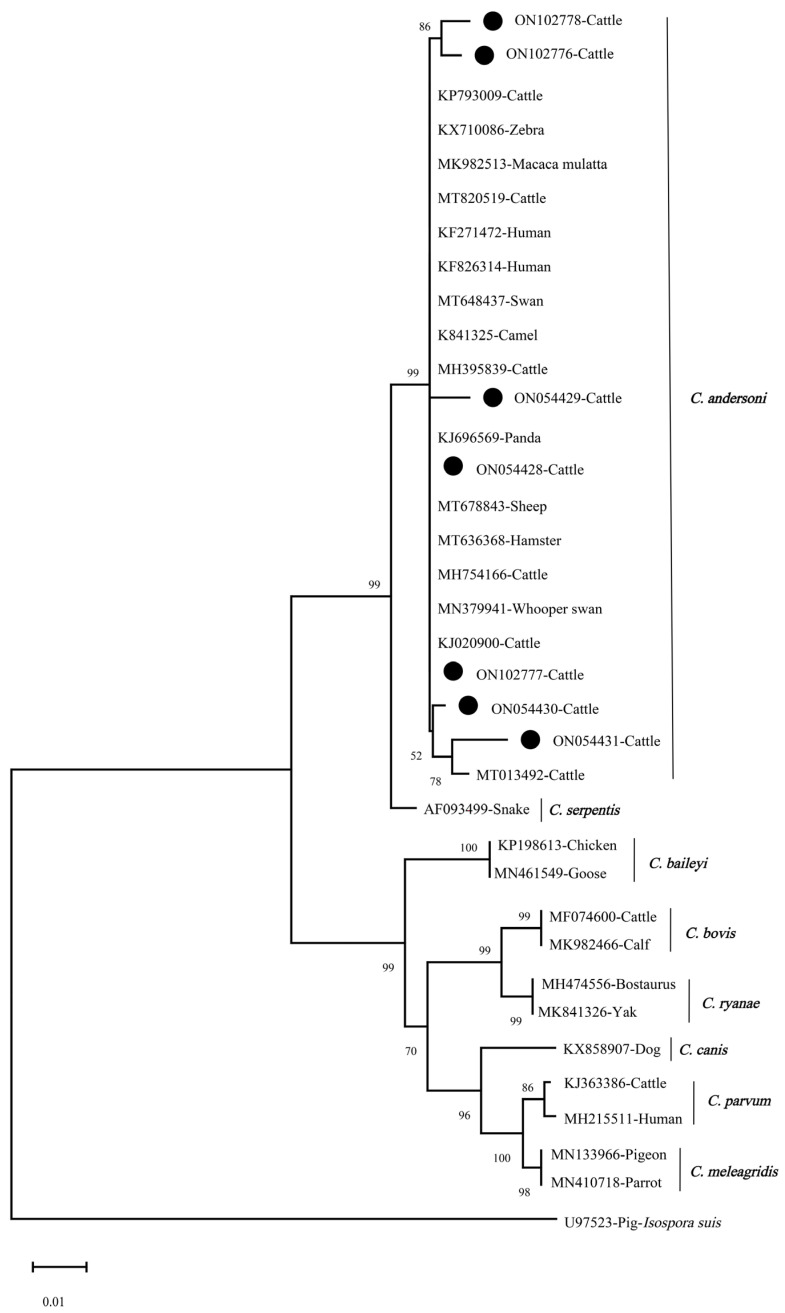
The Neighbor-Joining (NJ) method was used to analyze the phylogenetic relationship of *Cryptosporidium* species and/or genotypes identified in this study (marked with circles) and reported DNA sequences. Bootstrap value is shown when >50%.

**Figure 3:**
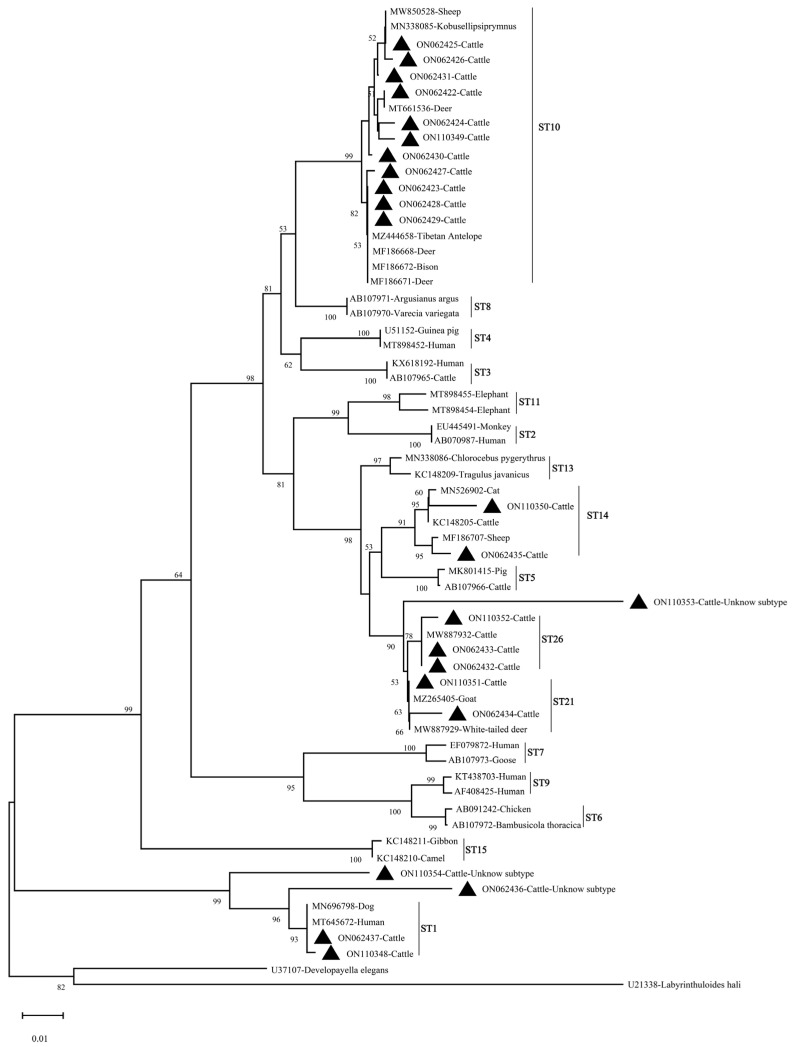
The Neighbor-Joining (NJ) method was used to analyze the phylogenetic relationship of *Blastocystis* subtypes identified in this study (marked with triangles) and reported DNA sequences. Bootstrap value is shown when >50%.

The nucleotide sequences of *Cryptosporidium* spp. and *Blastocystis* sp. obtained in this study were submitted to the GenBank database and the accession numbers are listed in Table 5. As shown in Figure 2 and Figure 3, the phylogenetic trees of *Cryptosporidium* spp. and *Blastocystis* sp. were constructed by using the Neighbor-Joining (NJ) method with the Kimura 2-parameter model, and the parameters were set to 1000 replicates (bootstrap) [14,22]. The *Cryptosporidium*-positive DNA sequences obtained in this study represented *C. andersoni* and are distributed in the same cluster in Figure 2. Specifically, the ON054428 sequence showed a 100% similarity to that of KX710086 (zebra), MH754166 (cattle), MK841325 (camel), MK982513 (Macaca mulatta), MT636368 (hamster) and MT648437 (swan), respectively. Furthermore, 103 *Blastocystis*-positive samples were identified to represent five known subtypes (ST1, ST10, ST14, ST21 and ST26) and three unknown subtypes of *Blastocystis* sp. using DNA sequences analysis, and the prevalent subtype in cattle in Shanxi Province was ST10 (n = 63). Notably, only two subtypes (ST10 and ST26) were detected in cattle in Shanyin County, and three unknown subtypes were found in cattle in Jishan County. The subtype ST10 was the dominant subtype in both beef and dairy cattle (Table 6); however, this subtype (ST10) was not detected in cattle in Qi County. A comparison of sequence homology showed that multiple DNA sequences of *Blastocystis* sp. identified in this study show 100% homology to reported DNA sequences in the GenBank database; for example, the sequence ON062425 (ST10) has 100% homology with MW850528 (sheep) and the sequence ON110351 (ST21) has 100% homology with MZ265405 (goat) and MW887929 (white-tailed deer). Also, the sequences ON062433 (ST26) and MW887932 (cattle) were 100% identical. Of the three unknown subtypes of *Blastocystis* sp. detected in this study, two of them were closely related to ST1, and the other was closely related to ST21 and ST26.

## 4. Discussion

An increasing number of studies have shown that *Cryptosporidium* spp. and *Blastocystis* sp. pose public health problems and cause economic losses to animal husbandry [23,24,25,26,27]. Prior to the present study, the prevalence of *Cryptosporidium* spp. and *Blastocystis* sp. in cattle in Shanxi Province remained unknown. In this study, the prevalence and population genetic structure of both *Cryptosporidium* spp. and *Blastocystis* sp. in cattle in Shanxi Province were analyzed for the first time. The overall prevalence of *Cryptosporidium* spp. and *Blastocystis* sp. in cattle in Shanxi Province was 2.9% (23/795) and 13.0% (103/795), respectively, as shown in Table 7 and Table 8, which, respectively, summarize the epidemiological data of *Cryptosporidium* spp. and *Blastocystis* sp. in cattle in China. The prevalence of *Cryptosporidium* spp. and *Blastocystis* sp. in cattle in Shanxi Province was lower than that in some provinces of China. We speculated that the oocysts of *Cryptosporidium* spp. shed intermittently and solid walls may have resulted in false negativity and the lower prevalence detected in this study. However, only seven published studies reported *Blastocystis* sp. in cattle in China, which is not sufficient to explain well the possible reasons for the large difference in *Blastocystis* sp. prevalence in cattle, and the assessment of the prevalence and virulence of *Blastocystis* sp. in cattle requires further research.

Regarding the difference in prevalence among reported areas of China, we speculated that the reasons for the statistically significant difference in the prevalence of *Cryptosporidium* spp. and *Blastocystis* sp. among different regions may be related to the geographical locations [13,18,22,28], regional environmental conditions [24,28,29], raising management patterns [14,30,31], immune status of hosts [13,28] and sampling methods [28]. For example, according to the reported surveys of *Cryptosporidium* spp. infection in many provinces of China and *Blastocystis* sp. in 31 countries in Asia, the geographical locations and environmental conditions are important influencing factors for differences in prevalence [26,32]. Studies on the prevalence of *Cryptosporidium* spp. and *Blastocystis* sp. in free-grazing cattle in China indicated that cattle management practices can cause large epidemiological differences [14,30]. In addition, previous reports have shown that differences in the prevalence of *Cryptosporidium* spp. and *Blastocystis* sp. in cattle are closely related to host immune status [6,28,33,34]. There are also many reports indicating that the sampling seasons [13,35,36], number of samples [14] and detection methods [22] also affect the results.

**Table 7:** The prevalence of *Cryptosporidium* spp. in cattle in China.

Area	No. Positive/Total	Prevalence (%)	Gene Locus	Years	References
Anhui Province	23/955	2.4	SSU rRNA	2018	[6]
Beijing city	21/822	2.6	SSU rRNA, *Gp*60	2014–2015	[28]
Gansu Province	59/1414	4.2	SSU rRNA, *Gp*60	2015	[37]
Shanghai city	303/818	37.0	SSU rRNA	2015–2016	[38]
Guangdong Province	63/1440	4.4	SSU rRNA	2016	[39]
Heilongjiang Province	27/423	6.4	SSU rRNA, *Gp*60	2019	[40]
Jiangxi Province	71/556	12.8	SSU rRNA	2019	[41]
Sichuan Province	40/278	14.4	SSU rRNA, *Gp*60	2016–2017	[42]
Taiwan	60/226	26.5	SSU rRNA	2017–2018	[43]
Yunnan Province	65/442	14.7	SSU rRNA, *Gp*60	2019–2020	[44]
Tibet	3/442	0.7	SSU rRNA	2016	[30]
Hebei Provinces and Tianjin city	10/1040	1.0	SSU rRNA, *Gp*60	2016	[45]
Xinjiang Uyghur Autonomous Region	70/1827	3.8	SSU rRNA	2013	[46]

**Table 8:** The prevalence of *Blastocystis* sp. in cattle in China.

Area	No. Positive/Total	Prevalence (%)	Gene Locus	Years	References
Qinghai Province	278/1027	27.1	SSU rRNA	2016–2017	[47]
Jiangxi Province	305/556	54.9	SSU rRNA	2019	[33]
Heilongjiang Province	14/147	9.5	SSU rRNA	2010–2016	[48]
Yunnan Province	119/987	12.1	SSU rRNA	2017–2018	a
Shaanxi Province	92/371	24.8	SSU rRNA	2018	a
Anhui Province	0/955	0	SSU rRNA	2018	a
Guangdong Province	9/479	1.9	SSU rRNA	2016	a

a: literature data published in China.

In this study, we found that the prevalence of *Cryptosporidium* spp. (7.0%, 19/273) and *Blastocystis* sp. (32.2%, 88/273) in Jishan County was significantly higher than that in Qi County and Shanyin County. Similarly, the previous investigation in the same study area showed that the highest prevalence of *Enterocytozoon bieneusi* was also detected in cattle in Jishan County [49]. Furthermore, we found that the prevalence of *Cryptosporidium* spp. and *Blastocystis* sp. in the three representative counties was gradually increased with increasing latitude. Villalobos-Segura et al. mentioned that latitude can affect the prevalence of parasites [50]. In addition, the prevalence of *Cryptosporidium* spp. and *Blastocystis* sp. may be affected by the sampling seasons. In the study of seasonal variations in the prevalence of *Cryptosporidium* spp. in cattle in the Xinjiang Uygur Autonomous Region, the highest prevalence was detected in summer [51]. Previous studies showed that the prevalence of *Blastocystis* sp. in pigs in Korea varies greatly according to the seasons, with the highest prevalence in winter and autumn [52].

Data on the prevalence of *Cryptosporidium* spp. in different gender groups of cattle in Shanxi Province indicated that the prevalence of *Cryptosporidium* spp. in male cattle (3.0%, 8/270) was higher than that in female cattle (2.9%, 15/525) with no statistically significant difference among gender groups (*p* = 0.933). However, no significant difference was observed between male cattle (10.2%, 13/128) and female cattle (4.1%, 6/145) in Jishan County. Similar to the results of this study, a survey of *Cryptosporidium* spp. infection in beef and dairy cattle in Peninsular Malaysia showed no significant correlation between *Cryptosporidium* spp. prevalence and gender factors [53]. However, Elmahallawy et al. from Egypt reported that the female calves were more susceptible to *Cryptosporidium* spp. than males [54]. Therefore, the role of gender as a risk factor for *Cryptosporidium* spp. infection needs to be elucidated through more research in the future. Regarding *Blastocystis* sp., the prevalence of *Blastocystis* sp. in the female cattle in this study was 15.0% (79/525), higher than that in the males (8.9%, 24/270). In addition, the present study indicated that a statistically significant difference was found in prevalence among different gender groups (*p* < 0.05), which was consistent with a previous report of *Blastocystis* sp. prevalence in pigs in China by Zou et al. [55]. Moreover, statistical analysis indicated that similar significant differences were observed in prevalence among different genders and types of cattle in Jishan County.

The highest prevalence of *Cryptosporidium* spp. was detected in cattle older than 18 months (3.2%, 10/314), followed by cattle aged 12–18 months (3.1%, 6/195) and younger than 12 months (2.4%, 7/286), but the difference was not statistically significant (*p* = 0.852). However, many studies showed that younger animals were more susceptible to *Cryptosporidium* spp. due to their compromised immune system [18,28,44,56]. In the farms sampled in this study, calves were separately fed in enclosures with more suitable temperature and humidity, which may be one of the reasons for the low prevalence of *Cryptosporidium* spp. in calves. The highest prevalence of *Blastocystis* sp. was found in cattle aged 12 to 18 months (15.4%, 30/195), followed by cattle older than 18 months (12.4%, 39/314) and younger than 12 months (11.9%, 34/286), but the difference was not statistically significant (*p* = 0.499). The results of *Blastocystis* sp. prevalence were similar to previous studies in cattle in China [57] and in Korea [58]. The correlation between the age of animals or humans and the prevalence of *Blastocystis* sp. has also been mentioned in other studies [59,60,61,62], but no fully consistent conclusions have been drawn due to the heterogeneity of sample sizes in different age groups.

In this study, the prevalence of *Cryptosporidium* spp. and *Blastocystis* sp. in beef cattle was higher than that in dairy cattle, and the prevalence was significantly different between the two types of cattle. In Jishan County, this difference in prevalence was more significant. Similar results have been found in previous studies in China’s Shaanxi Province and Korea. The prevalence of *Cryptosporidium* spp. in beef cattle and dairy cattle in Shaanxi Province was 4.5% (38/847) and 2.6% (32/1224), respectively [63]. In Korea, a survey showed that the prevalence of *Blastocystis* sp. in beef cattle was higher than that in dairy cattle [58]. We speculated that the differences in prevalence of *Cryptosporidium* spp. and *Blastocystis* sp. in beef cattle and dairy cattle in this study might be due to the differences in management patterns on different farms due to different types of cattle. In addition, differences in sanitation and parasite control measures across farms may also affect the prevalence of *Cryptosporidium* spp. and *Blastocystis* sp.. *C. andersoni* was the only *Cryptosporidium* species detected in cattle in this study. Recently, there have been relevant studies demonstrating that *C. andersoni* is the most prevalent species of *Cryptosporidium* spp. in cattle over one year, and it was also a common species of *Cryptosporidium* spp. detected in humans [64,65]. *C. andersoni* is also the most prevalent species in calves 2–12 months old in many regions of China, but not in other areas of the world. According to phylogenetic analysis, the *C. andersoni* identified in cattle was closely clustered with the *C. andersoni* isolated in humans (Figure 2). Five known subtypes (ST1, ST10, ST14, ST21 and ST26) and three unknown subtypes of *Blastocystis* sp. were detected in cattle in this study. Among these subtypes, ST10 was the predominant subtype in cattle worldwide [9,66]. Approximately 80% of ruminants were infected with the subtype ST10, posing a higher economic loss [57]. Subtype ST1 is one of nine major zoonotic subtypes infecting humans [9]. As shown in Figure 3, three unknown subtypes detected in this study were respectively clustered into a single branch, and their zoonotic potential still needs to be assessed. These findings suggested that effective measures must be taken to control and prevent *C. andersoni* infection in cattle in the study areas, and its zoonotic potential should also be taken into consideration in executing control strategies.

## 5. Conclusions

In this study, the overall prevalence of *Cryptosporidium* spp. in cattle in Shanxi Province was 2.9%, and all positive samples were identified as *C. andersoni*. The overall prevalence of *Blastocystis* sp. in cattle in Shanxi Province was 13.0%, five known subtypes and three unknown subtypes were detected, and the dominant subtype was ST10. There were significant differences in the prevalence of *Cryptosporidium* spp. and *Blastocystis* sp. in cattle from different regions. In addition, significant differences in the prevalence of *Blastocystis* sp. were found among the different genders. These findings not only enrich the knowledge of the population genetic structure of both parasites, but also provide baseline data for the control and prevention of these two zoonotic parasites in animals and humans.

## Figures and Tables

**Table 1:** Nested PCR amplification of the SSU rRNA gene in *Cryptosporidium* spp. and PCR amplification of the SSU rRNA gene in *Blastocystis* sp.

Parasite	Loci	Primer ID	Primer Sequences (5′-3′)	Annealing Temperature (°C)	Fragment Length (bp)	Reference
*Cryptosporidium*	SSU rRNA	F1	CCCATTTCCTTCGAAACAGGA	56		[19]
		R1	TTCTAGAGCTAATACATGCG			
		F2	AAGGAGTAAGGAACAACCTCCA	58	830	
		R2	GGAAGGGTTGTATTATTAGATAAAG			
*Blastocystis*	SSU rRNA	F1	ATCTGGTTGATCCTGCCAGT	65	613	[20]
		R1	GAGCTTTTTAACTGCAACAACG			

**Table 2:** The prevalence of *Cryptosporidium* spp. in cattle in Shanxi Province.

Factor	Categories	No. Samples	No. of Positive	Prevalence% (95% CI)	OR (95% CI)	*p*-Value
Region	Shanyi County	209	0	-	-	<0.001
	Qi County	313	4	1.3 (0–2.5)	1	
	Jishan County	273	19	7.0 (3.9–10.0)	5.8 (1.9–17.2)	
Gender	Female	525	15	2.9 (1.4–4.3)	1	0.933
	Male	270	8	3.0 (0.9–5.0)	1.0 (0.4–2.5)	
Age	M < 12	286	7	2.4 (0.7–4.2)	1	0.852
	12 ≤ M ≤ 18	195	6	3.1 (0.7–5.5)	1.3 (0.4–3.8)	
	M > 18	314	10	3.2 (1.2–5.1)	1.3 (0.5–3.5)	
Type	Dairy cattle	394	4	1.0 (0–2.0)	1	0.002
	Beef cattle	401	19	4.7 (2.7–6.8)	4.8 (1.6–14. 4)	
Total		795	23	2.9 (1.7–4.1)		

-: indicates no data available; M: month.

**Table 3:** The prevalence of *Blastocystis* sp. in cattle in Shanxi Province.

Factor	Categories	No. Samples	No. of Positive	Prevalence% (95% CI)	OR (95% CI)	*p*-Value
Region	Shanyin County	209	8	3.8 (1.2–6.4)	1.7 (0.6–4.9)	<0.001
	Qi County	313	7	2.2 (0.6–3.9)	1	
	Jishan County	273	88	32.2 (26.7–37.8)	20.8 (9.4–45.9)	
Gender	Female	525	79	15.0 (12.0–18.1)	1.8 (1.1–2.9)	0.014
	Male	270	24	8.9 (5.5–12.3)	1	
Age	M < 12	286	34	11.9 (8.1–15.6)	1	0.499
	12 ≤ M ≤ 18	195	30	15.4 (10.3–20.4)	1.3 (0.8–2.3)	
	M > 18	314	39	12.4 (8.8–16.1)	1.1 (0.6–1.7)	
Type	Beef cattle	401	79	19.7 (15.8–23.6)	3.8 (2.3–6.1)	<0.001
	Dairy cattle	394	24	6.1 (3.7–8.5)	1	
Total		795	103	13.0 (10.6–15.3)		

M: month.

**Table 4:** Distribution of the prevalence of *Cryptosporidium* spp. and *Blastocystis* sp. among risk factors in our study.

	Categories	Gender		Age			Type	
		Female	Male	M < 12	12 ≤ M ≤ 18	M > 18	Beef Cattle	Dairy Cattle
*Cryptosporidium* spp.	Shanyin County	0 (0/184)	0 (0/25)	0 (0/95)	0 (0/0)	0 (0/114)	0 (0/0)	0 (0/209)
	Qi County	1.0% (2/196)	1.7% (2/117)	2.6% (3/116)	1.1% (1/93)	0 (0/104)	0 (0/177)	2.9% (4/136)
	Jishan County	4.1% (6/145)	10.2% (13/128)	5.3% (4/75)	4.9% (5/102)	10.4% (10/96)	8.5% (19/224)	0 (0/49)
*Blastocystis* sp.	Shanyin County	4.3% (8/184)	0 (0/25)	8.4% (8/95)	0 (0/0)	0 (0/114)	0 (0/0)	3.8% (8/209)
	Qi County	3.6% (7/196)	0 (0/117)	2.6% (3/116)	0 (0/93)	3.8% (4/104)	0 (0/177)	5.1% (7/136)
	Jishan County	44.1% (64/145)	18.8% (24/128)	30.7% (23/75)	29.4% (30/102)	36.5% (35/96)	35.3% (79/224)	18.4% (9/49)

**Table 5:** The nucleotide sequence data of *Cryptosporidium* spp. and *Blastocystis* sp. obtained in this study.

Parasite	Type	Subtypes	Accession Numbers
*Cryptosporidium*	Beef cattle	-	ON054428, ON054429, ON054430, ON054431
	Dairy cattle	-	ON102776, ON102777, ON102778
*Blastocystis*	Beef cattle	ST1	ON062437
		ST10	ON062422, ON062423, ON062424, ON062425, ON062426, ON062427, ON062428, ON062429, ON062430, ON062431
		ST14	ON062435
		ST21	ON062434
		ST26	ON062432, ON062433
		Unknown subtype	ON062436
	Dairy cattle	ST1	ON110348
		ST10	ON110349
		ST14	ON110350
		ST21	ON110351
		ST26	ON110352
		Unknown subtype	ON110353
		Unknown subtype	ON110354

**Table 6:** Subtype distribution of *Blastocystis* sp. in cattle in three representative counties of Shanxi Province.

Factors	Categories	No. Samples	No. of Positive	Subtype (n)
Region	Shanyi County	209	8	ST10 (7), ST26 (1)
	Qi County	313	7	ST1 (1), ST10 (2), ST14 (2), ST21 (1), ST26 (1)
	Jishan County	273	88	ST1 (3), ST10 (54), ST14 (6), ST21 (3), ST26 (19), unknown subtypes (3)
Type	Beef cattle	401	79	ST1 (3), ST10 (48), ST14 (6), ST21 (2), ST26 (19), unknown subtype (1)
	Dairy cattle	394	24	ST1 (1), ST10 (15), ST14 (2), ST21 (2), ST26 (2), unknown subtypes (2)

ST: subtype.

## Data Availability

The data sets supporting the results of this article have been submitted to GenBank and accession numbers are shown in the article.
